# Early intra‐abdominal hypertension: A reliable bedside prognostic marker for severe acute pancreatitis

**DOI:** 10.1002/jgh3.12393

**Published:** 2020-08-04

**Authors:** Kailash C Kurdia, Santhosh Irrinki, Arun V Chala, Ashish Bhalla, Rakesh Kochhar, Thakur D Yadav

**Affiliations:** ^1^ Department of General Surgery Postgraduate Institute of Medical Education and Research Chandigarh India; ^2^ Department of Internal Medicine Postgraduate Institute of Medical Education and Research Chandigarh India; ^3^ Department of Gastroenterology Postgraduate Institute of Medical Education and Research Chandigarh India

**Keywords:** intra‐abdominal hypertension, intra‐abdominal pressure, prognostic marker, severe acute pancreatitis

## Abstract

**Background and Aim:**

Severe acute pancreatitis (SAP) is commonly associated with intra‐abdominal hypertension (IAH). This acute increase of intra‐abdominal pressure (IAP) may be attributed to early organ dysfunction, leading to an increased morbidity and mortality. To assess the incidence of raised IAH and its correlation with other prognostic indicators and various outcomes in SAP.

**Methods and Results:**

This was a prospective observational study in patients of SAP between July 2009 and December 2010. All patients of SAP who were admitted to the hospital within 2 weeks of onset of pain were included in the study. A total of 35 patients with SAP were included in the study. Among these, 25 (71.4%) were males. All our patients had raised IAP; however, IAH was present in 51.4% (18/35). Patients with IAH were found to have a higher APACHE II score (88.9 *vs* 5.9%; *P* < 0.001), infectious complications (72.2 *vs* 5.9%; *P* < 0.001), circulatory failure (88.9 *vs* 0%; *P* < 0.001), and respiratory failure (100 *vs* 41.2%; *P* < 0.001). All the eight (22.8%) patients who succumbed to sepsis had IAH. Patients with IAH were found to have a significantly longer intensive care unit (ICU) stay (17.72 *vs* 12.29 days) and in‐hospital stay (24.89 *vs* 12.29 days).

**Conclusion:**

IAH is a good negative prognostic marker in SAP, seen in up to 51.4%. IAH was found to have a significant negative impact on the outcome in terms of increased mortality, morbidity, in‐hospital stay, and ICU stay among the patients of SAP.

## Graphical abstract

This study has proven the correlation of raised intra‐abdominal pressure in severe acute pancreatitis with poor outcome. Hence, serial monitoring of intra‐abdominal pressure in severe acute pancreatitis should be performed, and appropriate measures should be taken when it is present.

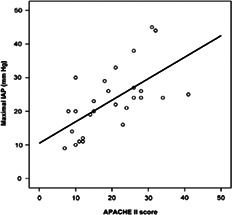

## Introduction

Severe acute pancreatitis (SAP) is a potentially life‐threatening disease and accounts for 20% of all pancreatitis.^1^ Overall mortality of acute pancreatitis (AP) is around 5%; however, it can reach up to 20–30% in SAP. The mortality of infected necrosis ranges from 47 to 69% among patients who develop multiorgan dysfunction syndrome (MODS).^2^, ^3^, ^4^, ^5^, ^6^

Mortality in AP has a bimodal peak. About 50% of patients die within first 2 weeks following a sequalae of inflammatory mediators leading to MODS. This disease is caused by excessive leukocyte activation, leading to the release of secondary inflammatory mediators [Interleukins (IL) ‐ IL‐1α, IL‐6, IL‐8, IL‐10, tumour necrosis factor (TNF‐α), platelet‐activating factor, nitric oxide (NO), arachidonic acid metabolites], resulting in massive inflammatory response, contributing to the induction of systemic inflammatory response syndrome (SIRS). Mortality after 2 weeks is usually due to septic complications.^7^, ^8^, ^9^, ^10^, ^11^, ^12^

Evidence suggests that, in addition to SIRS, untreated abdominal compartment syndrome (ACS) is one of the contributing factors to mortality in the early phase of SAP. Intra‐abdominal pressure (IAP) ranges between 0 and 5 mm of Hg and varies with the respiratory cycle.^13^, ^14^ According to the World Society on Abdominal Compartment Syndrome (WSACS), intra‐abdominal hypertension (IAH) is defined as the sustained increase in IAP above 12 mm of Hg and ACS as IAP above 20 mm of Hg with new‐onset organ failure with or without low abdominal perfusion pressure (APP).^15^ However, even IAP less than 15 mm of Hg may cause organ dysfunction.^16^, ^17^

Massive fluid resuscitation in the early course of the disease, combined with the severe inflammatory process in the retroperitoneum, could contribute to visceral edema, leading to increased IAP. This acute increase of IAP in severe cases may lead to early organ dysfunction and ACS.^18^ Paralytic ileus, upper gastrointestinal tract obstruction by pancreatic collection, and reduced abdominal wall compliance due to edema are other factors aggravating IAH in AP.^19^

There are several studies reporting the incidence and consequences of IAH in intensive care patients, but in SAP, the literature is limited. The present study was undertaken to assess the significance of IAP in SAP and its relation to various other mortality indicators in AP.

## Methods

All patients who were admitted to the Department of General Surgery and Medical Gastroenterology with diagnosis of SAP from July 2009 to December 2010 were enrolled in the study. A diagnosis of SAP was made based on revised Atlanta criteria in 2012. All patients with SAP who presented within 2 weeks from the onset of symptoms were included in the study. Patients who denied consent and who presented more than 2 weeks after the onset of symptoms were excluded.

The study is approved by the Institutional Ethics Committee as per institutional protocol.

### *Intra‐abdominal pressure*

IAP was monitored by measuring intravesical pressure in the supine position at the end of expiration at a 4‐hourly interval by using Foleys catheter with a three‐way stop cock. IAP was measured on the day of admission to the hospital every 4 h, and the values of peak IAH were noted. A sustained increase in IAP was determined if at least three consecutive standardized measurements were elevated. Patients were divided into two groups (A and B) based on the absence and presence of IAH, respectively (IAH‐IAP >12 mm of Hg), based on the peak IAP values.

### *Management*

All patients were managed according to the standard protocol, which included nasogastric decompression, intravenous fluids, analgesics, and other supportive measures. Antibiotic selection during the management was performed based on the evidence of sepsis and the availability of culture and sensitivity reports. Mechanical ventilation and vasopressor support were provided as and when indicated. Nutritional support was preferably enteral unless contraindicated. APACHE II score was calculated at admission. Organ failure during the first 7 days of admission was determined using SOFA score. Surgery was performed in the early period when other conservative measures to decrease IAH failed and at a later period in patients who did not respond to step up approach.

### *Statistical analysis*

Descriptive statistics were used. Data were expressed as mean ± SD or mean (range) as appropriate. All statistical calculations were performed using the IBM Statistical Package for Social Sciences (SPSS) II version 22.0.0.0 software for Windows version 1.60. Nonparametric tests (Mann–Whitney *U* test) and linear regression analysis were used to evaluate ordinal data such as maximal IAP. The Chi square test was used for analysis of nominal data. Statistical significance was set as *P* < 0.05.

## Results

Of the 35 patients included, 25 were males, and 10 were females. Etiology was alcohol in 19 (54.3%) patients, gall stone disease (GSD) in 10 (28.6%) patients, idiopathic disease in 4 patients, and hyperparathyroidism and endoscopic retrograde cholangio‐pancreatography (ERCP) in 1 patient each. The overall mortality was 22.85%. IAH was present in 51.4% (18/45) of the study population.

**Figure 1:**
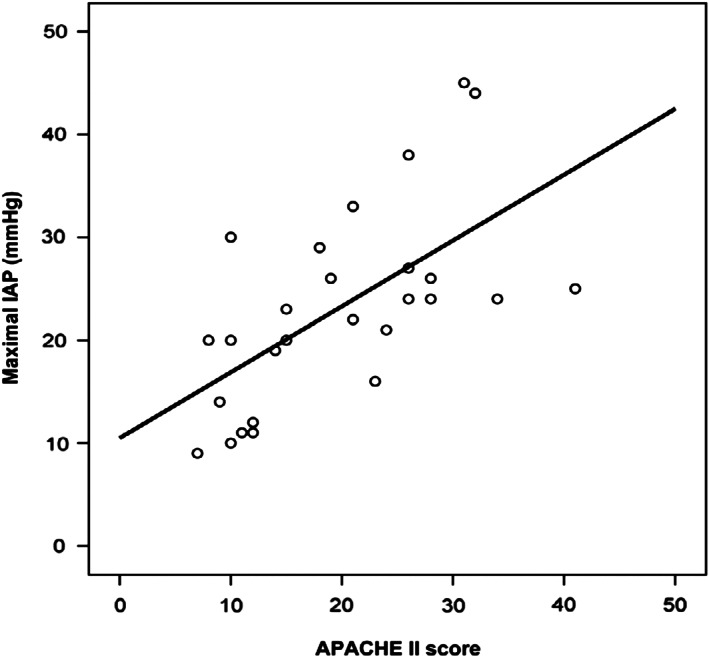
Correlation between maximal intra‐abdominal pressure and APACHE II. IAP, intra‐abdominal pressure.

On comparison of demographics, etiology, modified CT severity index, various complications, and mortality, IAH was found to be higher in female and older patients, although this was not statistically significant. APACHE II score (Fig. 1), infectious complications, organ failure, and mortality were higher in patients with IAH (Tables 1 and 2). IAP of more than 20 mmHg was seen in three (8.5%) patients who eventually deteriorated. One of the three required percutenous drainage (PCD) for decompression but later succumbed to multiorgan failure; the remaining two patients required surgery—one for decompression and the other for infected necrosis. Both patients died eventually due to organ failure and septic shock, respectively.

**Table 1:** Gender distribution and mean age of those with intra‐abdominal pressure (IAH) and without IAH in severe acute pancreatitis

Characteristics	IAH present (18)	IAH absent (17)	*P* value
Male	10 (40%)	15 (60%)	0.060
Female	8 (80%)	2 (20%)	
Gender ratio (Male: Female)	1:1	3.5:1	
Age ± SD (in years)	42.89 ± 13.86	31.59 ± 11.35	0.013

**Table 2:** Comparison of variables between the two groups

Characteristic	With IAH	Without IAH	*P* value
Total	18 (51.4%)	17 (48.6%)	
Male	10	15	0.06
Female	8	2	
Age SD (in years)	42.89 ± 13.86	31.59 ± 11.35	0.013
Etiology			
Alcohol	6	13	
GSD	7	3	0.089
Hyperparathyroidism	1	0	
ERCP	1	0	
Idiopathic	4	1	
APACHE‐II			
<8	2	16	<0.001
>8	16	1	
Modified CTSeverity Index (CTSI)			
7–8	13	17	<0.001
>8	5	0	
Necrosis			
<50%	13	16	0.117
>50%	5	1	
CRP (mean ± SD) in mg/dL	126.89 ± 45.3	54.12 ± 36.79	<0.001
SOFA score (day 1–7)			
<5	0	10	
5–10	13	7	<0.001
>10	5	0	
APACHE II (at Admission)			<0.001
<8	2	16	
>8	16	1	
Infections at admission	1	0	0.34
Infections during hospitalization	13	1	<0.001
Organ failure			
Renal	18	16	0.486
Cardiovascular	16	0	<0.001
Respiratory	18	7	<0.001
Mortality	8	0	0.003

CRP, C reactive protein; CTSI, Computerised tomography severity index; ERCP, endoscopic retrograde cholangio‐pancreatography ; GSD, gall stone disease; IAH, intra‐abdominal hypertension.

**Figure 2:**
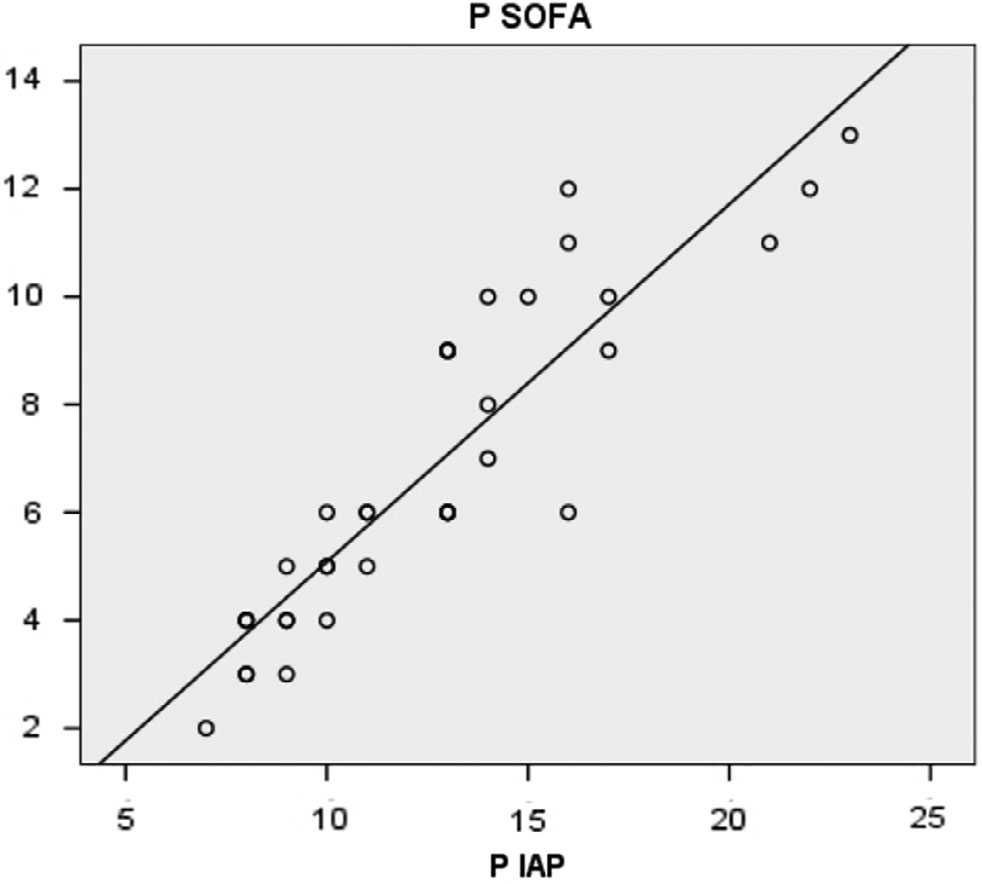
Maximal SOFA score (days 1–7) and maximal intra‐abdominal pressure (IAP) (days 1–5). (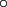), Observed; (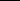), linear.

Requirement of percutaneous drainage, total parenteral nutrition, and surgical management were found to be high in those patients with IAH. In addition to IAH, maximal IAP was also found to be in linear correlation with maximal SOFA score during the first week (Fig. 2), as well as in predicting the need for parenteral nutrition, surgery, and mortality during the hospital stay (Tables 3 and 4).

**Table 3:** Table showing correlations of intra‐abdominal hypertension (IAH) with need for percuteneous drainage (PCD), total paranteral nutrition (TPN), and surgery

Management	With IAH	Without IAH	*P* value
PCD	6	2	0.129
TPN	9	0	0.001
Surgery	5	0	0.019
Mortality	8	0	0.003

**Table 4:** Table showing the relation of mean intra‐abdominal hypertension (IAP) and maximal IAP in predicting the need for percutenous drainage (PCD), parenteral nutrition (TPN), surgery, and overall outcome in severe acute pancreatitis

	Mean IAP ± 2SD (95% CI)	Maximum IAP ± 2SD (95% CI)	*P* value
Parenteral Nutrition required			
Yes	11.82 ± 1.75	15.56 ± 2.55	0.05
No	9.32 ± 3.51	11.50 ± 4.07	0.016
PCD requirement			
Yes	10.47 ± 1.35	13.25 ± 1.98	0.628
No	9.81 ± 3.71	12.33 ± 4.57	0.588
Surgery			
Yes	14.44 ± 4.97	17.80 ± 4.55	<0.001
No	9.22 ± 2.34	11.67 ± 3.38	0.008
Outcome			
Survivors	8.74 ± 1.87	11.00 ± 2.74	<0.001
Nonsurvivors	14.07 ± 3.93	17.75 ± 3.77	<0.001

CI, confidence interval.

## Discussion

IAH is caused by several clinical conditions, for example, hemoperitoneum, bowel obstruction, intestinal edema, peritonitis, pelvic trauma, and surgery (aortic surgery and abdomen closure under tension). AP is one of the main causes of IAH of retroperitoneal origin.^20^, ^21^, ^22^

According to the current definition of IAH, the reported incidence of IAH and ACS in AP is 60 and 27%, respectively.^22^, ^23^, ^24^, ^25^, ^26^ In the present study, the incidence of IAH and ACS was 51.4 and 8.5%, respectively. Several mechanisms have been proposed for the development of IAH. Severe pancreatitis usually presents with an enlarged pancreas with inflammation and fluid collections in the retroperitoneum. In the beginning, it is localized, only to progress later to the whole retroperitoneum, including the root of mesentery. Necrosis further aggravates the problem. Other factors associated with SAP that contribute to IAH are ascites, visceral edema, ileus, and gastric dilatation due to collections or mechanical obstruction of the duodenum. Fluid resuscitation further plays an important role in the development of IAH due to capillary leakage as a result of a diffuse intra‐abdominal inflammatory process. IAH itself initiates a vicious cycle due to decreasing intestinal perfusion and compromised oxygen delivery.^27^

Several studies established that infectious complications are higher in those patients who had IAH, reaching up to 60% in patients who had ACS.^23^, ^28^, ^29^, ^30^ The present study also confirms that infectious complications were very high (77.8%) in patients with IAH. Mean and maximal IAP were significantly higher in those patients who required surgery, as well those who died. Several prognostic factors, such as APACHE II, modified CTSI, CRP, and SOFA score were also found to be higher in the present study, which was comparable to the available evidence.^31^, ^32^, ^33^, ^34^ Ke *et al*. compared APP and IAP to determine severity and complications in AP and concluded that IAP is more valuable as an early measure for the evolution and complication of SAP.^33^ Waele *et al*. studied the incidence of IAH and organ dysfunction in 44 (27 patients underwent IAP measurement) patients admitted in the ICU with diagnosis of SAP. Their result showed that IAH was frequently (78%) present and was associated with a high incidence of organ dysfunction, as well as high mortality. They suggested that the direct causal relationship between IAH and organ dysfunction was not proven in patients with SAP, and surgical decompression should not be routinely performed as it was associated with higher mortality.^35^ Similarly, in the present study, the incidence of IAH was 51.4%, and ACS was found only in three patients; most of these patients were managed conservatively, and only two patients underwent surgical decompression in those three patients with ACS, and all three patients died. IAH is associated with organ dysfunction in AP, and to some extent, it is reversible and preventable through early management. This study advocates the need for frequent measurement of IAP at least every 4 h or whenever the clinical condition of the patient deteriorates to avoid unwanted outcomes such as IAH and ACS. Resuscitation in the early period of pancreatitis should judicious, and several recent trials showed that a combination of crystalloids and colloids is better than crystalloid alone to prevent IAH.^36^, ^37^, ^38^ Several nonsurgical interventions have been tried to decrease IAP, such as nasogastric tube decompression, percutaneous drainage of intraperitoneal fluid, and neuromuscular blockers for the short term, which were reported to be beneficial.^39^, ^40^ Other interventions for removal of extra fluid by loop diuretics, extracorporeal technique, and hemodialysis have also been tried with variable outcomes. Loop diuretics should be used with caution because acute kidney injury is one of the first organ dysfunctions and may further accentuate renal failure.^27^ In a recent study, Chen *et al*. found that a combination of nonsurgical interventions was able to reduce IAP in 7 of 20 patients with ACS and avoided decompressive laparotomy in each of them.^23^ In the present study, one patient underwent percutaneous drainage for ACS; although IAP was decreased by 8 mmHg, the patient later succumbed to septic shock. Although surgery should be avoided in the early phase of SAP, few acute complications require early intervention, such as ACS not responding to conservative management, bowel ischemia, perforation, and severe bleeding not managed by radiological intervention. Several methods were used for surgical decompression, and the most common of these are midline laparotomy followed by bilateral subcostal incision for full‐thickness laparotomy and short horizontal skin incision to perform subcutaneous linea alba fasciotomy (SLAP) with the peritoneum left intact.^41^, ^42^, ^43^ All these methods have been shown to have variable outcome. In the present study, three (8.5%) patient with ACS required intervention. One underwent percutaneous drainage for decompression, and the remaining two patients underwent laparotomy for decompression and infected necrosis, respectively. In spite of interventions, these three patients could not be saved.

### *Limitations*

A small sample size, the lack of information regarding the role of fluid resuscitation, and the effect of decompression in modulating the course of SAP that were not within the purview of this study were the probable limitations of this study. A multivariate analysis was also not performed.

In summary, our study, in accordance with other studies, shows that increased IAP is common in SAP and is significantly correlated with the various other indicators of severity in SAP. The overall incidence of IAH in SAP was around 51.4%. It also has a negative impact on the outcome of the disease in terms of increased mortality and morbidity, along with the increase in the requirement of hospital stay and intensive care support. In fact, all the patients who succumbed in this study had evidence of IAH. It is thus necessary to measure IAP routinely as it is an important parameter associated with the course, management, and outcome of SAP.
